# Photo Quiz: Skin scraping of persistent lesion reveals the main course

**DOI:** 10.1128/jcm.00220-24

**Published:** 2024-11-13

**Authors:** Christopher Attaway, Jennifer Andrasko, Anisha Misra

**Affiliations:** 1 University of Vermont Medical Center, Burlington, Vermont, USA; 2 Pathology and Laboratory Medicine Institute, Cleveland Clinic, Cleveland, Ohio, USA; Mayo Clinic Minnesota, Rochester, Minnesota, USA

## PHOTO QUIZ 

**Fig 1:**
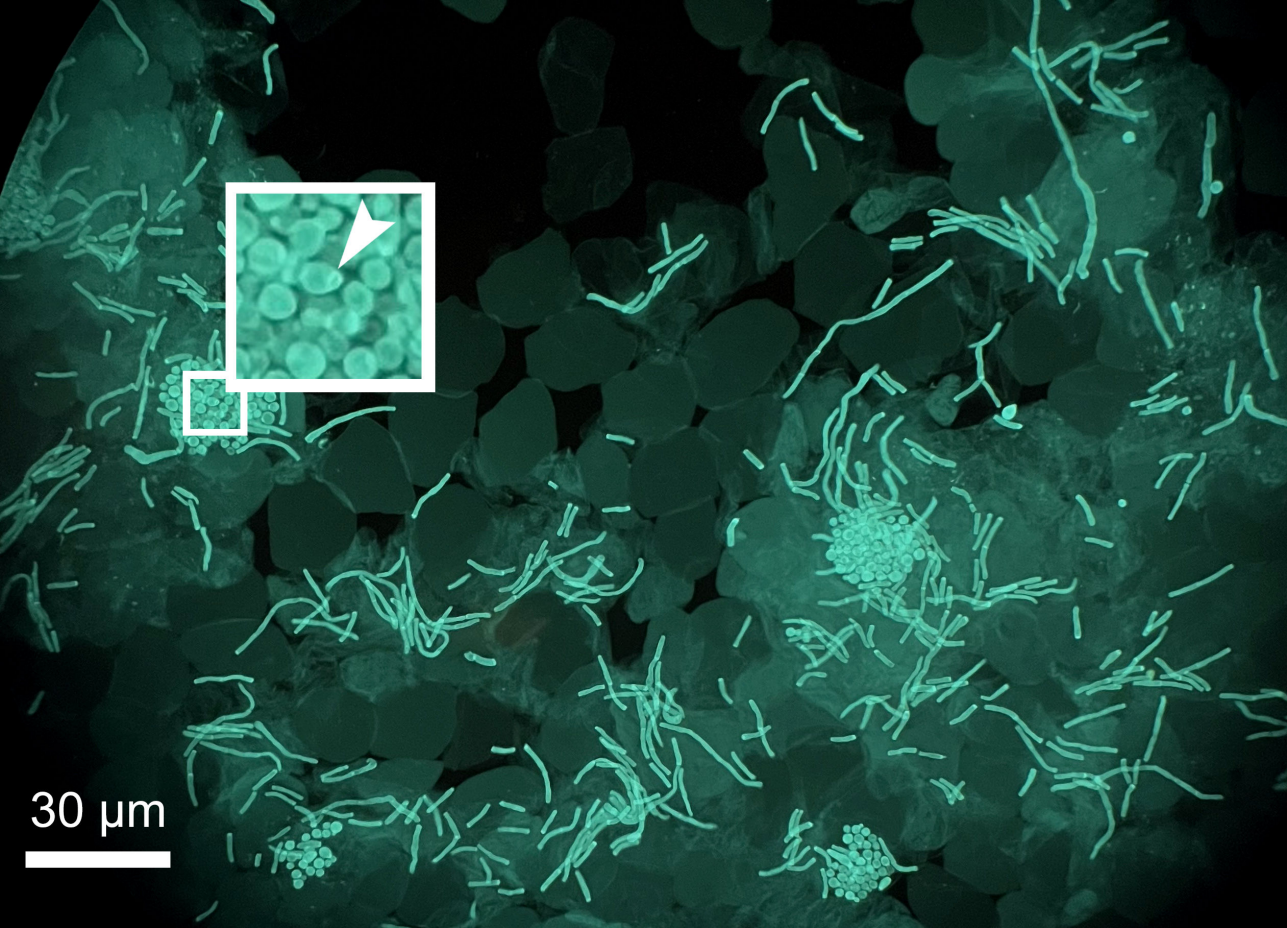
KOH-calcofluor white stained skin scraping specimen revealing clusters of budding yeast, some with a broad collarette (arrow) with associated hyphal forms.

A 32-year-old female with a past medical history notable for eczema and systemic lupus erythematosus presented to an outpatient dermatologist’s office with a hypopigmented, painless large patch on her back. Upon physical exam, the patch comprised finely scaled, disrupted superficial epithelium with an erythematous, irregular border. The patient revealed that she had been prescribed a topical steroid cream several weeks ago; however, her skin had not improved. A superficial scraping of the erythematous border of the patch was sent to the microbiology laboratory for evaluation. A KOH calcofluor white stain showed clusters of round, budding yeast, some with broad collarettes or “shoulders” and short rectangular hyphal forms (Fig. 1). What is the most likely diagnosis?

## ANSWER TO PHOTO QUIZ

*Malassezia* species are basidiomycete yeasts that cause the superficial fungal infection clinically known as tinea versicolor (or pityriasis versicolor). The classic microscopic appearance, described as “spaghetti and meatballs,” is seen only in clinical specimens (skin scrapings and biopsies), with the yeast forms being the “meatballs” and the hyphal forms the “spaghetti” (1). Visualization of these features on potassium hydroxide (KOH) preparation, calcofluor stains, and histopathology raises suspicion for *Malassezia* species. The budding sites of the yeast cells have a flat collarette, while the opposite end is rounded.

The collarette—visible by Gram or lactophenol blue stains—helps distinguish *Malassezia* from *Candida albicans* and other yeasts with hyphal forms (e.g., *Trichosporon* spp.) (2). Communicating suspicion for *Malassezia* to the clinical microbiology laboratory is necessary for successful culture as these organisms require supplemented or specialized media. The genus *Malassezia* represents numerous lipophilic commensal species, a subset of which are implicated most frequently as the causative agent of tinea versicolor: *M. globose*, *M. sympodialis*, *M. sloofiae*, and *M. furfur*. These lipophilic species should be plated on media overlayed with sterile olive oil or Tween. *Malassezia pachydermatis* is the exception and does not require lipid supplementation (3, 4). Alternatively, there are commercially available media, Dixon’s agar or Leeming-Notman medium and their modified counterparts, which are ideal for the isolation of *Malassezia* species; there is also a chromogenic agar (CHROMagar Malassezia, Paris, France) available.

*Malassezia* species are a commensal constituent of human skin flora. Through an unknown mechanism, *Malassezia* species that are pathogenic produce hyphal forms (5). Identification to the species level is not usually required for superficial mycosis as the treatment is identical for all *Malassezia* species—a topical triazole or terbinafine cream, though oral antifungals are an option in severe or recurrent infection (1). In the case of fungemia, species identification is required as systemic treatment will be warranted, and susceptibility profiles are not necessarily predictable between species (6).

Clinically, as seen in this patient, *Malassezia* species form superficial, large, coalescing, scaly, painless patches on the trunk and proximal limbs but can be found anywhere on the body. Macules or patches may be hypo- or hyperpigmented (5). A Wood’s lamp examination can reveal a yellow-green to copper-orange fluorescence, though *Malassezia* does not always fluoresce (1, 3, 7). *Malassezia* species are capable of more severe infections, such as folliculitis or seborrheic dermatitis (8). Interestingly, studies have shown that multiple *Malassezia* species can be isolated from a single patient (9). An important consideration is that as *Malassezia* species are skin flora constituents, their lipophilic nature places patients receiving parenteral nutrient (lipid) supplementation at a higher risk of *Malassezia* fungemia from colonized catheters (1).

In our laboratory, the specimen was plated on mycobiotic and potato dextrose agar containing chloramphenicol (PDACH) overlayed with olive oil. Plates were incubated at 30°C. Scant colonies were observed only on the PDACH with olive oil overlay after 7 days. Although the original and subcultured plates were held for 28 days, no better growth was observed despite providing fatty acids in media. Malassezia are fastidious yeast that are sensitive to transport and culture conditions. Inability to accurately imitate their natural habitat can significantly impact their viability. Due to the limited number of colonies, only MALDI-ToF was performed; neither Bruker MALDI Biotyper (Bruker Daltonics Incorporated, Massachusetts, USA) nor Vitek MS MALDI (bioMérieux, Marcy-l’ Étoile) was able to provide identification for our isolate, despite the organisms being represented in both databases. Remaining colony was sent for D2, and ITS sequencing revealed 100% sequence identity with both *M. furfur* and *M. globose* species. Different yeast budding characteristics can help distinguish between these entities (broad base with *M. furfur* and narrow base with *M. globose*), but these features could not be confidently characterized on the initial calcofluor preparation. The organism was unable to be cultured for additional studies that may have helped to differentiate and determine a species-level identification, such as *M. furfur’s* ability to grow at high Tween concentrations (Tween 80) and *M. globose*’s precipitate production on Dixon’s agar. Additionally, sequencing of the intergenic spacer 1 region of the rRNA gene could potentially provide discrimination between *Malassezia* species, although this is not performed by our laboratory (10). Inability to obtain sufficient growth highlights a common laboratory challenge in providing definitive identification and the need for culture-independent diagnostic methods (11). The isolate was finalized as “*Malassezia* species (most closely resembling to *M. globosa/furfur*).” The patient was treated with nystatin-triamcinolone topical ointment, and the infection resolved after 1 month.
